# Coinfecting parasites can modify fluctuating selection dynamics in host–parasite coevolution

**DOI:** 10.1002/ece3.6373

**Published:** 2020-08-30

**Authors:** Otto Seppälä, Curtis M. Lively, Jukka Jokela

**Affiliations:** ^1^ Institute of Integrative Biology ETH Zürich Zürich Switzerland; ^2^ Department of Aquatic Ecology Eawag Dübendorf Switzerland; ^3^ Research Department for Limnology University of Innsbruck Mondsee Austria; ^4^ Department of Biology Indiana University Bloomington IN USA

**Keywords:** coevolution, coinfection, Red Queen dynamics, resource competition, virulence

## Abstract

Genetically specific interactions between hosts and parasites can lead to coevolutionary fluctuations in their genotype frequencies over time. Such fluctuating selection dynamics are, however, expected to occur only under specific circumstances (e.g., high fitness costs of infection to the hosts). The outcomes of host–parasite interactions are typically affected by environmental/ecological factors, which could modify coevolutionary dynamics. For instance, individual hosts are often infected with more than one parasite species and interactions between them can alter host and parasite performance. We examined the potential effects of coinfections by genetically specific (i.e., coevolving) and nonspecific (i.e., generalist) parasite species on fluctuating selection dynamics using numerical simulations. We modeled coevolution (a) when hosts are exposed to a single parasite species that must genetically match the host to infect, (b) when hosts are also exposed to a generalist parasite that increases fitness costs to the hosts, and (c) when coinfecting parasites compete for the shared host resources. Our results show that coinfections can enhance fluctuating selection dynamics when they increase fitness costs to the hosts. Under resource competition, coinfections can either enhance or suppress fluctuating selection dynamics, depending on the characteristics (i.e., fecundity, fitness costs induced to the hosts) of the interacting parasites.

## Graphical abstract

Coinfections by multiple parasite species are a rule in nature and could affect host–parasite coevolutionary dynamics by altering infection dynamics. This simulation model shows that coinfections can enhance fluctuating selection dynamics between hosts and parasites when interactions between coinfecting species increase the fitness costs to the hosts. Under resource competition, however, coinfections can either enhance or suppress fluctuating selection dynamics.

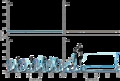

## 1 INTRODUCTION

Hosts and parasites are expected to coevolve antagonistically (reviewed in Lively, 2001; Thompson, 1994). Parasites obtain at least part of their resources from hosts (Price, 1980), which reduces host fitness (i.e., parasites are virulent). Hosts, on the other hand, are under selection to minimize these costs by preventing/eliminating infections using defense mechanisms such as immune function (reviewed in Janeway, Travers, Walport, & Shlomchik, 2005; Söderhäll, 2010). Host–parasite coevolution can lead to different outcomes including fluctuating selection dynamics (see Gandon, Buckling, Decaestecker, & Day, 2008; Woolhouse, Webster, Domingo, Charlesworth, & Levin, 2002). Of particular interest in this model is that parasites can adapt to common host types, leading to frequency‐dependent selection. Specifically, parasite genotypes that can successfully infect the most common host types have a selective advantage, which increases their frequency in the parasite population. This increase can intensify selection against common host types and drive their frequencies down, simultaneously allowing some previously rare host types that are not under strong parasite‐mediated selection to become common. This variation in selective pressure can then lead to fluctuations in host and parasite genotype frequencies over time (Hamilton, 1980).

Fluctuating selection dynamics have received vast theoretical attention (e.g., Galvani, Coleman, & Ferguson, 2003; Hamilton, 1980; Lively, 2010b; May & Anderson, 1983) and empirical evidence supporting their occurrence in natural populations is available from a few study systems (Decaestecker et al., 2007; Dybdahl & Lively, 1998; Jokela, Dybdahl, & Lively, 2009). Fluctuating selection dynamics are, however, expected to occur only when specific requirements for host and parasite characteristics are fulfilled (e.g., high fitness costs of infection to the hosts; see Lively, 2010b). Various environmental/ecological factors such as resource availability, ambient temperature, and coinfecting parasites can, however, strongly affect host and parasite performance (e.g., host and parasite fecundity [Guinnee & Moore, 2004; Lello, Boag, & Hudson, 2005; Paull & Johnson, 2011; Seppälä, Liljeroos, Karvonen, & Jokela, 2008], host survival [Brown, Loosli, & Schmid‐Hempel, 2000; Krist, Jokela, Wiehn, & Lively, 2004; Louhi, Sundberg, Jokela, & Karvonen, 2015; Seppälä et al., 2008]). Furthermore, ecological factors can alter genetic specificity in determining parasite infection success (Sadd, 2011; Zouache et al., 2014), which could affect the ability of parasites to induce frequency‐dependent selection on their hosts. Therefore, ecological factors contributing to and creating variation in the outcome of host–parasite interactions could be important in determining the potential for fluctuating selection dynamics in host–parasite coevolution.

In this study, we focused on the possible effects of coinfecting parasite species on fluctuating selection dynamics. Host populations typically maintain a community of parasites (reviewed in Holmes & Price, 1986), and individual hosts are often simultaneously infected with more than one parasite species (e.g., Fountain‐Jones et al., 2019; Lello, Boag, Fenton, Stevenson, & Hudson, 2004; Rellstab, Louhi, Karvonen, & Jokela, 2011). Coinfections often lead to higher fitness costs to the hosts when compared to single infections (e.g., Johnson & Hoverman, 2012; Lello et al., 2005). Furthermore, coinfecting parasites can interact with each other through competition for the shared host resources (Bashey, Hawlena, & Lively, 2012; Patrick, 1991), direct interference (Bashey et al., 2012; Massey, Buckling, & R. ffrench‐Constant., 2004), and/or host immune function (Adams, Anderson, & Windon, 1989; Brady, O'Neill, Dalton, & Mills, 1999). Therefore, coinfections may also alter parasite survival and reproductive output (e.g., Johnson & Hoverman, 2012; Lello et al., 2005; Randall, Cable, Guschina, Harwood, & Lello, 2013). Importantly, these effects could modify selection between hosts and parasites, depending on the composition of the coinfecting parasite community.

Earlier, in a related field of parasite‐mediated selection for sex, coinfections by multiple parasite species have been suggested to be important, as the combined effect of coinfecting parasites could reduce host fitness enough for sexual reproduction to be favored by selection (Hamilton, Axelrod, & Tanese, 1990). The same could enhance fluctuating selection dynamics if several parasite species, each having only a weak negative impact on host fitness, track the host genotypes in a frequency‐dependent manner. Many parasites, however, do not show strict genetic specificity to their hosts, but can infect different host genotypes and even species (reviewed in Schmid‐Hempel, 2011). Nonetheless, such generalist parasites could alter the performance of hosts and their genetically specific parasites through the above‐mentioned interaction mechanisms and this way contribute to fluctuating selection dynamics.

Here, we formally examined if and how coinfections by a genetically nonspecific generalist parasite could contribute to fluctuating selection dynamics between a host and its genetically specific parasite using numerical simulations. We modeled host population dynamics and parasite epidemiology under three different scenarios: (a) when hosts coevolve with a single parasite species that must genetically match the host to infect, (b) when hosts are also exposed to a genetically nonspecific parasite species that increases fitness costs to the hosts in coinfections (coinfection does not alter parasite performance compared with single infections), and (c) when coinfecting parasites compete for the shared host resources (coinfection does not increase host fitness costs compared with the mean of single infections). We focused on these two coinfection scenarios because of their contrasting effects on host and parasite fitness, as well as their likely commonness in nature (e.g., some parasites use the same and some different host resources). Our model shows that the presence of coinfecting parasites can strongly impact fluctuating selection dynamics. Coinfections can enhance fluctuating selection dynamics when they increase fitness costs to the hosts. Under resource competition, coinfections can either enhance or suppress fluctuating selection dynamics, depending on the characteristics (i.e., fecundity, fitness costs induced to the hosts) of the interacting parasites.

## 2 MODEL

Our simulation is based on the epidemiological model by Lively (2010b), which incorporates two ecologically relevant aspects that are likely to be important. First, it considers host fitness to be density‐dependent. Second, it does not define the probability of infection as simply a function of the frequency of the matching parasite genotype, but considers the numerical feedbacks from the parasite population. This is important because epidemiological dynamics can influence host–parasite coevolution (e.g., Gokhale, Papkou, Traulsen, & Schulenburg, 2013; MacPherson & Otto, 2018). The model by Lively (2010b) assumes discrete generations (one generation per time step) in which the resistance of sexually reproducing individuals to infection is determined by two loci each having three alleles (giving nine genotypes). We used similar discrete time steps, but because our model is not connected to the question of sexual/asexual reproduction we simplified the original model by assuming that hosts reproduce clonally. We did not reduce the number of host genotypes from nine to two, although only two are commonly used in other theoretical studies (e.g., Gokhale et al., 2013; MacPherson & Otto, 2018; Song, Gokhale, Papkou, Schulenburg, & Traulsen, 2015). We chose to use nine host genotypes to increase the ecological relevance of the model (see Dybdahl & Lively, 1998; Jokela et al., 2009; Little & Ebert, 1999) and because the pilot runs of the simulation indicated an increased role of stochasticity in determining host population dynamics when only two genotypes were used.

To examine the potential role of interactions between coinfecting parasites on fluctuating selection dynamics, our model assumed that hosts can be exposed to two different horizontally transmitted parasite species. Parasite species A is genetically specific: Each genotype is only able to infect one of the host clones (i.e., a matching allele model [see Agrawal & Lively, 2002] that reflects self‐nonself recognition in invertebrates [Frank, 1993; Luijckx, Fienberg, Duneau, & Ebert, 2013]). All host clones are, however, exposed to all parasite genotypes, but parasites that do not match the host are eliminated by host defenses. On the other hand, parasite species B is genetically nonspecific and able to infect all host genotypes. We chose these two parasite types because genetic specificity in determining the outcome of a host–parasite interaction (here parasite A) is required for fluctuating selection dynamics, but many parasite species do not show strict genetic specificity (here parasite B) being able to infect a broad range of different host genotypes and even different host species (reviewed in Schmid‐Hempel, 2011). Therefore, owing to the commonness of coinfections in nature, these parasite types are likely to often interact.

We examined fluctuating selection dynamics under two different scenarios of interactions between the parasite species. In the first scenario, we assumed that in coinfections, parasites use different host resources, but exploit hosts with the same efficiency as in single infections. Therefore, coinfections increase fitness costs to the hosts. We chose this increase to be multiplicative rather than additive. Multiplicative increase in fitness costs is possible, for example, if one parasite species reduces the host's ability to detect resources and the other parasite reduces the efficiency of resource use after detection. Additive effects of coinfections on host fitness are also likely in nature. These interaction types are, however, conceptually similar. Our choice to use multiplicative effects is based on their stronger impacts on hosts, which makes it easier to evaluate whether interactions that reduce host fitness could potentially alter fluctuating selection dynamics. In this scenario, coinfections did not affect parasite performance.

In the second scenario, we assumed that coinfecting parasites compete for the shared host resources. For simplicity, we did not consider variation in parasite within‐host growth rate. Furthermore, we did not specifically model possible priority effects in resource use in sequential coinfections (e.g., Clay, Dhir, Rudolf, & Duffy, 2019; Hoverman, Hoye, & Johnson, 2013). This was because we conducted modeling at the population level. Thus, we assumed that, on average, each coinfecting species has access to 50% of the available host resources. We also assumed that each species uses these resources with the same efficiency as the resources available in single infections. Therefore, reduced availability of host resources for each parasite species in coinfections reduces parasite fecundity to one half of their reproductive output in single infections. Consequently, the fitness cost of coinfection to the hosts was equal to the mean of the costs experienced in single infections.

In our model, the density of *i*th host genotype at time point *t* + 1 (*Gʹ_i_*) was

(1) $$G_{i}^{\prime }=W_{I(A)}\left(P_{i(A)}-P_{i(\text{AB})}\right)G_{i}+W_{I(B)}\left(P_{i(B)}-P_{i(\text{AB})}\right)G_{i}+W_{I(\text{AB})}P_{i(\text{AB})}G_{i}+W_{U}\left(1-P_{i(A)}-P_{i(B)}+P_{i(\text{AB})}\right)G_{i}$$

where *W_I_*_(A)_ is host fitness when infected with parasite A only, *W_I_*_(B)_ is host fitness when infected with parasite B only, *W_I_*_(AB)_ is host fitness when infected with both parasite species, *W*_*U*_ is host fitness when uninfected, *P_i_*_(A)_ is the probability of infection with parasite A, *P_i_*_(B)_ is the probability of infection with parasite B, *P_i_*_(AB)_ is the probability of infection with both parasite species, and *G_i_* is the density of the *i*th host genotype at time point *t*. Note that the probabilities of single infections and coinfection follow the probability theory, *P_i_*_(AB)_ being *P_i_*_(A)_ × *P*_(B)_. Each element in the summation represents the contribution of different host types (infected with parasite A only, infected with parasite B only, infected with both parasite species, uninfected) to the next generation.

Host fitness was density‐dependent following the formulation by Maynard Smith and Slatkin (1973):

(2a) $$W_{U}=\frac{b_{U}}{1+aN}$$

(2b) $$W_{I(A)}=\frac{b_{I(A)}}{1+aN}$$

and

(2c) $$W_{I(B)}=\frac{b_{I(B)}}{1+aN}$$

for uninfected hosts and hosts infected with parasite A and parasite B (single infections), respectively. In these equations, *b*_*U*_ is the reproductive output of uninfected hosts when competition is absent, *b_I_*_(A)_ is the reproductive output of hosts infected with parasite A when competition is absent, *b_I_*_(B)_ is the reproductive output of hosts infected with parasite B when competition is absent, *a* is a parameter that scales the effect of the total host density on host reproductive output, and *N* is the total host population density. Note that the effects of infections and host population density on host reproductive output can arise from both differences in host fecundity and survival. Here, we used the same *a* for uninfected and infected individuals, hence infection reduced host fecundity, but it did not make infected individuals more sensitive to competition. When defined this way, the cost of infection to the hosts induced by parasites A (*C*_A_) and B (*C*_B_) become

(3a) $$C_{A}=1-\frac{b_{I(A)}}{b_{U}}$$

and

(3b) $$C_{B}=1-\frac{b_{I(B)}}{b_{U}}$$

for all host densities, and the population dynamics are stable for all values of *b* (Doebeli & de Jong, 1999; Lively, 2010b). Thus, in coinfection scenario one (parasites use different host resources), host fitness under coinfection (*W_I_*_(AB)_) becomes

(4a) $$W_{I(\text{AB})}=W_{U}\times \left(1-C_{A}\right)\times \left(1-C_{B}\right)$$

whereas in scenario two (parasites compete for the shared host resources), it becomes

(4b) $$W_{I(\text{AB})}=W_{U}\times \frac{\left[\left(1-C_{A}\right)+\left(1-C_{B}\right)\right]}{2}$$

The probability of infection for each host genotype and each parasite species was modeled according to the Poisson distribution. We assumed that parasite propagules are released into the environment and that each contact between a matching host and parasite results in infection. Furthermore, we assumed that each infection with one or more propagules of the same parasite species leads to a similar infection in the host (i.e., exposure to multiple propagules of the same parasite does not lead to a more intense infection). Thus, the probability of infection with parasite species A for each individual of the *i*th host genotype (*P_i_*_(A)_) at time point *t* + 1 was.

(5) $$P_{i(A)}=1-e^{\left[-\frac{\beta_{A}G_{iI\left(A\right)}+\beta_{\text{AB}}G_{iI\left(\text{AB}\right)}}{N^{\prime }}\right]}$$

where *β*_A_ is the number of parasite propagules produced by each singly infected host, *β*_AB_ is the number of parasite A propagules produced by each coinfected host (scenario 1: *β*_AB_ = *β*_A_; scenario 2: *β*_AB_ = *β*_A_/2), *G_iI_*_(A)_ is the density of hosts of genotype *i* that are infected with parasite A only, *G_iI_*_(AB)_ is the density of coinfected hosts of genotype *i*, and *Nʹ* is the total host population density at time point *t* + 1. The term (*β*_A_ *G_iI_*_(A)_ + *β*_AB_ *G_iI_*_(AB)_)/*Nʹ* gives the Poisson mean number of exposures per host. The number of produced parasite propagules (i.e., the numerator in the equation) is divided by the total host population density in the next generation because host individuals are exposed to all parasite genotypes but only those that genetically match the host lead to infections. The exponential term gives the probability of not being exposed by a matching parasite genotype and hence remaining uninfected. Similarly, the probability of infection with parasite species B for each host individual in the population (*P*_B_) was

(6) $$P_{B}=1-e^{\left[-\frac{\beta_{B}N_{I\left(B\right)}+\beta_{\text{BA}}N_{I\left(\text{AB}\right)}}{N^{\prime }}\right]}$$

where *β*_B_ is the number of parasite propagules produced by each singly infected host, *β*_BA_ is the number of parasite B propagules produced by each coinfected host (scenario 1: *β*_AB_ = *β*_B_; scenario 2: *β*_AB_ = *β*_B_/2), *N_I_*_(B)_ is the density of hosts that are infected with parasite B only, and *N_I_*_(AB)_ is the density of coinfected hosts.

We divided each run of the simulation into two phases. During the first 2000 generations, only the genetically specific parasite species (i.e., parasite A) was present in the host population. The initial host population density was 18,000, and the frequency of each host clone *i* at the beginning of the simulation was defined as

(7) $$f_{i}=\frac{R_{i}}{\sum_{i=1}^{9}R_{i}}$$

where *R* is a random value derived from a uniform distribution. One of the individuals in each host clone was defined to be infected at the beginning. Reproductive output of uninfected hosts (*b*_*U*_) was 20 (after Lively, 2010b). Parasite fecundity (*β*_A_) was examined between the values zero and 60 in intervals of 0.2, and the proportional reduction in host fitness (i.e., cost of infection [*C*_A_]) between the values zero and one in intervals of 0.1. We examined the whole range of potential fitness effects of infection on hosts because host exploitation rate varies among parasite species and host sensitivity to infection can depend on the specific organ the parasite infects. We allowed migration into the host population to prevent hosts and parasites from local extinction. Specifically, the probability that an uninfected host entered the population was 0.10 for each clone in each generation. The probability that an infected host entered the population was 0.02 for each clones (after Lively, 2010b).

In each simulation run, we examined the epidemiological and fluctuating selection dynamics during the last 100 generations because pilot runs showed that the dynamics became predictable during the first 1,500 generations. We took the following measures from the host population: mean infection prevalence (proportion of host individuals infected [Bush, Lafferty, Lotz, & Shostak, 1997]), mean host population density, variation (variance) in host population density, and mean change in clone frequencies between consecutive generations (range: 0–1). The last measure quantifies fluctuating selection dynamics and is affected both by the period and the amplitude of coevolutionary cycles: When frequencies of clones change a lot between the time points, the frequency and the amplitude of the resulting cycles increase. Note that measuring the cycle period and the amplitude directly (see Greenspoon & Mideo, 2017) is not feasible in our study because of high variation in the dynamics among individual cycles (see Section 3). It is also important to note that our measure for fluctuating selection dynamics is continuous and that the strength of coevolutionary cycling may change gradually over the examined parameter space. Therefore, our analysis does not aim to define strict limits for regions of the parameter space in which fluctuating selection dynamics are observed.

In the second phase of each simulation run, the host population was invaded by nine individuals (one per clone) infected with the genetically nonspecific parasite species B. In scenario one, this parasite used different host resources than parasite A, which increased the costs of infection to the hosts. In scenario two, parasites competed for the shared host resources (the fitness cost of coinfection to the hosts was equal to the average of costs in single infections). After the invasion of the second parasite, we let the simulation run for another 2000 generations and examined the above‐mentioned epidemiological and coevolutionary dynamics for the last 100 generations (probabilities of uninfected and infected immigrants were the same as in phase 1 for both parasite species). Additionally, we quantified the mean prevalence of parasite B in the host population. We examined the effects of the presence of the coinfecting parasite B using different levels of its fecundity (*β*_B_: 1.2, 1.4, 1.8, 2.6) and costs induced to the hosts (*C*_B_: 0.1, 0.3, 0.5, 0.7, 0.9) in all possible combinations. We chose the levels of fecundity based on the mean prevalence that such a parasite would have in a host population when infecting it alone (for *β*_B_ = 1.2, prevalence was 31%; for *β*_B_ = 1.4, prevalence was 51%; for *β*_B_ = 1.8, prevalence was 73%; for *β*_B_ = 2.6, prevalence was 90% when *C*_B_ ≤ 0.7 and 86% when *C*_B_ = 0.9). This way we could examine the effects of coinfecting parasites that have different frequencies in the host population. We chose the levels of fitness costs induced to the hosts to cover a broad range of parasites with different potential to control for host population density. If infecting the host population alone, a mildly harmful genetically nonspecific parasite would have only a weak impact on the host population density even if it was common (for *β*_B_ = 2.6 and *C*_B_ = 0.1, mean host population density was ≈17,200), whereas a highly harmful parasite would strongly reduce host population density (for *β*_B_ = 2.6 and *C*_B_ = 0.9, mean host population density was ≈3,500). Note that the host clone frequencies did not fluctuate over time in the runs including only a genetically nonspecific parasite species and the observed minor changes arose owing to immigration. We ran the simulation for each combination of the examined parameter values 20 times and report the mean of those runs when presenting the results.

## 3 RESULTS

### 3.1 Epidemiological and fluctuating selection dynamics in one host–one parasite interaction

**FIGURE 1:**
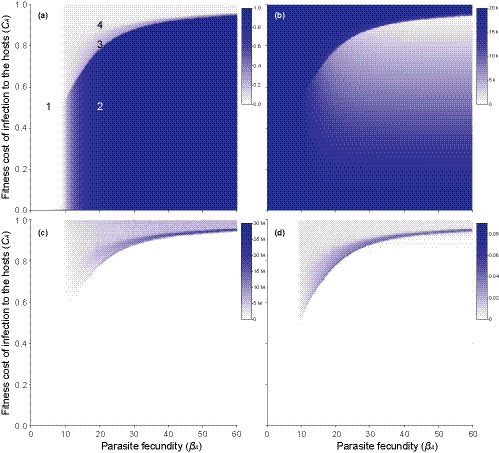
(a) Mean infection prevalence, (b) mean host population density, (c) variance in host population density across generations, and (d) mean change in host clone frequencies over time (i.e., fluctuating selection dynamics; range: 0–1) examined across different levels of parasite fecundity and fitness costs of infection to the hosts during the last 100 generations of the phase one of the simulation (coinfecting genetically nonspecific parasite not present). After Lively (2010b), the reproductive output of uninfected hosts (*b*_*U*_), the probabilities for uninfected and infected hosts to enter the population (same for all clones) were chosen to be 20, 0.10, and 0.02, respectively. Numbers 1–4 in the first panel refer to combinations of parameter values for which examples of dynamics of host clone frequencies in individual runs of the simulation are presented in Figure 2

**FIGURE 2:**
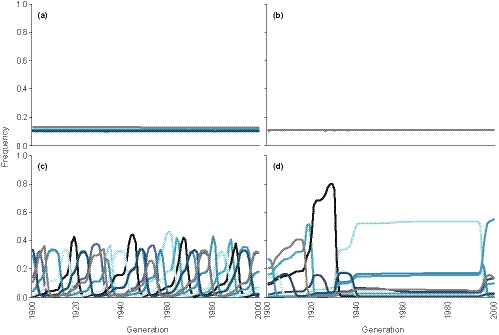
Examples of the dynamics of host clone frequencies (nine clones) in individual runs of the simulation representing regions of parameter space with different epidemiological and/or coevolutionary dynamics (numbers 1–4 in Figure 1a). (a) Parasite‐induced fitness costs to the hosts (*C*_A_) and parasite fecundity (*β*_A_) are 0.5 and 5, respectively (region 1 in Figure 1a), (b) *C*_A_ and *β*_A_ are 0.5 and 20, respectively (region 2 in Figure 1a), (c) *C*_A_ and *β*_A_ are 0.8 and 20, respectively (region 3 in Figure 1a), (d) *C*_A_ and *β*_A_ are 0.9 and 20, respectively (region 4 in Figure 1a)

The examined parameter space over different levels of parasite fecundity and host fitness costs showed separation into regions with distinct epidemiological and coevolutionary features (Figure 1). When parasite fecundity *β*_A_ was nine (i.e., equal to the number of host clones) or lower, parasites did not spread in the host population (the completely white region 1 in Figure 1a), they did not control for the host population density (Figure 1b,c), and did not lead to fluctuations in host clone frequencies (Figures 1d and 2a). The inability of the parasite to spread in the host population was most likely because when, on average, 8/9 of the parasite propagules die when contacting nonmatching host genotypes the basic reproductive number of the parasite is less than one (Lively, 2010a).

When parasite fecundity was higher than the number of host clones in the population, the parasites spread. The subsequent effects on the host population dynamics, however, depended on the level of fitness costs of infection to the host (Figure 1). When the costs were low to moderate, increasing parasite fecundity rapidly led to high infection prevalence (dark blue region 2 in Figure 1a) and infections kept the host population density constantly at a reduced level (Figure 1b,c). Under these conditions, host clone frequencies did not fluctuate over time (Figures 1d and 2b). The above dynamics, however, changed when the fitness costs of infection increased, unless parasite fecundity was very high. When the parasite reduced host fitness, for example, by 80% and parasite fecundity was 20 (or any other values in the light blue region 3 in Figure 1a), parasite prevalence remained low to moderate (Figure 1a) and it did not strongly reduce the average host population density (Figure 1b). However, host population density (Figure 1c) and clone frequencies (Figures 1d and 2c) showed strong temporal fluctuations, the latter indicating fluctuating selection dynamics.

**FIGURE 3:**
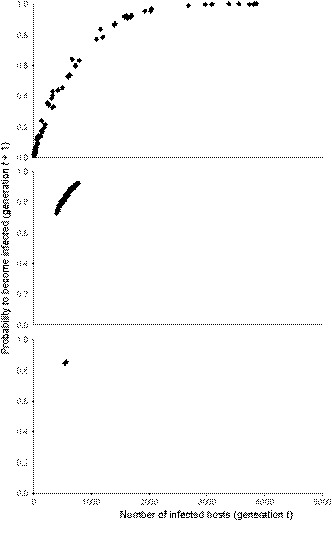
The relationship between the number of infected host individuals of one host clone in generation *t* and the probability of the individuals of the same clone to become infected in generation *t* + 1 during the last 100 generations of one simulation run. (a) Parasite‐induced fitness costs to the hosts (*C*_A_) and parasite fecundity (*β*_A_) are 0.78 and 20, respectively. (b) *C*_A_ is 0.775 and *β*_A_ is 20. (c) *C*_A_ is 0.77 and *β*_A_ is 20. These examples are chosen to demonstrate the abrupt change from the fluctuating selection dynamics to stable infection dynamics when fitness costs of infection to the hosts decrease

The above shift from stable control of the host population density by the parasite to fluctuating selection dynamics when the fitness costs of infection to the hosts increased was due to changed transmission potential of the parasite. Under fluctuating selection dynamics, parasites drove the density of the host clones that they infect temporarily close to zero (Figure 2c). During those periods, the absolute number of infected host individuals, as well as parasite propagules that can infect hosts in the next generation, was low. Thus, the infection risk was also low (Figure 3a) and the parasite could not reach a high and constant prevalence. When the parasite did not strongly reduce host fitness, the minimum host density remained always higher. Therefore, the number of parasite propagules and the infection risk in the next generation were higher (Figure 3b,c). Thus, higher fitness costs of infection to the hosts were required for fluctuating selection dynamics when parasite fecundity increased (Figure 1d).

When the fitness costs of infection were even higher than above (e.g., 90% reduction in host fitness) and parasite fecundity was 20 (or any other values in the almost white region 4 in Figure 1a), the coevolutionary dynamics changed again (Figures 1d and 2d). Now, alterations in host clone frequencies were detected periodically, but these reflected extinction and recolonization dynamics of host clones, rather than fluctuating selection dynamics (see generations 1900–1940 in Figure 2d). This was because highly harmful parasites drove the host clones quickly to extinction. These clones recovered only after the immigration of new individuals into the population. However, those clones were pushed back to extinction quickly after the parasite invaded the population owing to the immigration of an infected host individual. Furthermore, clone frequencies were stable when parasites were extinct (see generations 1940–1990 in Figure 2d).

### 3.2 Fluctuating selection dynamics in one host–two parasites interactions

**FIGURE 4:**
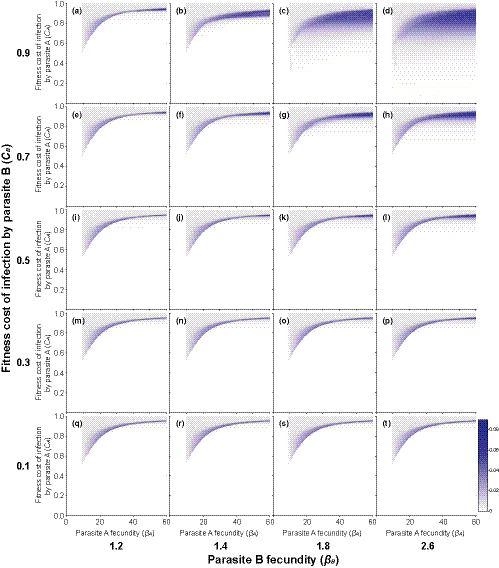
Mean change in host clone frequencies over time (i.e., fluctuating selection dynamics; range: 0–1) across different levels of fecundity and fitness costs induced to the hosts by the genetically specific parasite species A during the last 100 generations of the phase two of the simulation when the coinfecting genetically nonspecific parasite species B that uses different host resources was present in the host population. Coinfection leads to a multiplicative increase in fitness costs to the hosts. Plots a‐t show the results for different levels of parasite B fecundity and fitness costs it induces to the hosts. After Lively (2010b), the reproductive output of uninfected hosts (*b*_*U*_), the probabilities for uninfected and infected hosts to enter the population (both parasite species, same for all clones) were chosen to be 20, 0.10, and 0.02, respectively

**FIGURE 5:**
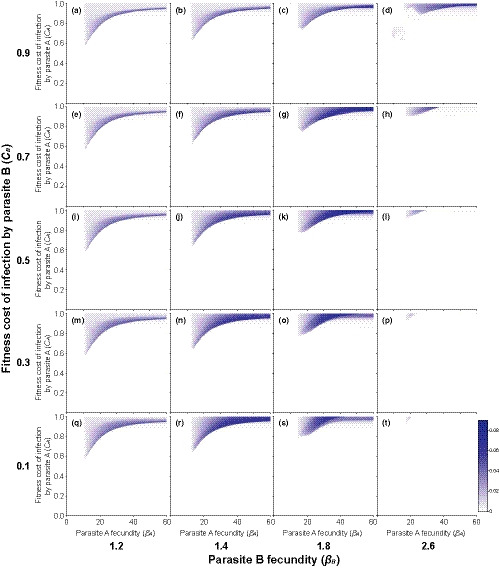
Mean change in host clone frequencies over time (i.e., fluctuating selection dynamics; range: 0–1) across different levels of fecundity and fitness costs induced to the hosts by the genetically specific parasite species A during the last 100 generations of the phase two of the simulation when the coinfecting genetically nonspecific parasite species B that uses the same host resources was present in the host population. Coinfection leads to competition for the shared host resources and thus reduces parasite fecundity. Plots a–t show the results for different levels of parasite B fecundity and fitness costs it induces to the hosts. After Lively (2010b), the reproductive output of uninfected hosts (*b*_*U*_), the probabilities for uninfected and infected hosts to enter the population (both parasite species, same for all clones) were chosen to be 20, 0.10, and 0.02, respectively

The above coevolutionary dynamics were affected by the presence of the coinfecting genetically nonspecific parasite species (Figures 4 and 5). When the parasites used different host resources, thus increasing the fitness costs of infection to the hosts (scenario 1), coinfections enhanced fluctuating selection dynamics (Figure 4). This effect was, however, clearly detected only when the genetically nonspecific parasite species was highly harmful to its hosts (see Figure 4 for *C*_B_ being equal to or higher than 0.7). Furthermore, the effect got stronger with the increasing fecundity and thus the prevalence of the coinfecting species in the host population. In this scenario, fluctuating selection dynamics were observed across a wider range of different levels of fitness costs induced to the hosts by the genetically specific parasite compared with the single host–single parasite interaction (see the above section). The expansion was toward lower levels of fitness costs of infection. This was because the increased host fitness costs in coinfections reduced parasite transmission potential (see the mechanism in Figure 3), which prevented the genetically specific parasite from reaching a very high prevalence, thus allowing fluctuating selection dynamics.

When the two parasite species competed for the shared host resources (scenario 2), the effect of coinfections on fluctuating selection dynamics became different (Figure 5). Now, even higher costs of infection to the hosts induced by the genetically specific parasite species were needed for fluctuating selection dynamics to occur than in the single host–single parasite interaction (see the above section). However, a broader range of different levels of fitness costs to the hosts led to fluctuating selection dynamics. The occurrence of fluctuating selection dynamics when the costs of infection to the hosts were very high was possible because temporal extinctions of host and parasite genotypes were less frequent in this scenario compared with the single host–single parasite interaction. This effect was strongest when the fecundity of the genetically nonspecific parasite was moderate (see Figure 5 for *β*_B_ being 1.4 and 1.8), and the costs of infection to the hosts induced by the genetically nonspecific parasite were low to moderate (Figure 5). This is likely to be because such a coinfecting species would frequently interact with the genetically specific parasite but not limit fluctuations in densities of host clones as it would not strongly reduce host population size. However, when the fecundity of the coinfecting genetically nonspecific parasite species was high (*β*_B_ = 2.6; i.e., it was common in the host population) coinfections suppressed fluctuating selection dynamics (Figure 5) as even maximal reduction in host fitness induced by the genetically specific parasite could not lead to fluctuating selection dynamics.

## 4 DISCUSSION

Individual hosts are often infected with multiple parasite species and genotypes that interact (reviewed in Holmes & Price, 1986; Read & Taylor, 2001). These interactions are expected to be important for key evolutionary processes in host–parasite interactions including selection for higher virulence (reviewed in Alizon, de Roode, & Michalakis, 2013) and the maintenance of genetic polymorphism in parasite traits (reviewed in Seppälä & Jokela, 2016). Additionally, increased fitness costs to the hosts owing to multiple infections have been proposed to strengthen parasite‐mediated selection for sex (Hamilton et al., 1990). In this study, we expanded the investigation on evolutionary consequences of coinfections to their potential role in host–parasite coevolutionary dynamics. We formally examined if and how fluctuating selection dynamics between coevolving host and parasite populations could be modified by the presence of another parasite species that does not track its hosts in a genetically specific manner but (a) increases fitness costs of infection to the hosts or (b) competes for the shared host resources with the coevolving parasite. We found that interactions that increase fitness costs to the hosts can enhance fluctuating selection dynamics. However, resource competition among parasites can both enhance and suppress coevolution, depending on the characteristics (i.e., fecundity, harmfulness to the hosts) of the interacting parasites.

Our results on fluctuating selection dynamics in a single host–single parasite interaction are in line with earlier findings suggesting that coevolutionary fluctuations are likely to take place only with certain combinations of parasite fecundity and fitness costs of infection to the hosts (see e.g., Lively, 2010b; May & Anderson, 1983). Specifically, fluctuating selection dynamics required high costs of infection to the hosts (*C*_A_ > 0.6, depending on parasite fecundity) that induce strong parasite‐mediated selection. However, very high costs led to host population dynamics that were mainly driven by temporal extinctions of the host and parasite genotypes. The requirement for “sufficiently high” costs of infection could limit the occurrence of fluctuating selection dynamics in natural systems. This is because many host species may not be frequently exposed to parasites that are harmful enough. The above‐mentioned idea of Hamilton et al. (1990) that suggests that simultaneous infections with multiple parasite species, each having a weak negative impact on their hosts, could lead to a total reduction in host fitness that is high enough to favor sexual reproduction could also hold for fluctuating selection dynamics. This would be the case when several mildly harmful parasite species simultaneously track the host genotypes in a frequency‐dependent manner.

Many parasites, however, do not show strict genetic specificity to their hosts. Although such parasites cannot induce frequency‐dependent selection on their hosts, they could still contribute to fluctuating selection dynamics by being part of the coinfecting parasite community. This is because coinfecting parasites often interact (e.g., Adams et al., 1989; Bashey et al., 2012; Patrick, 1991). Our study suggests that such interactions can be highly important for host–parasite coevolution. Furthermore, they can both enhance and suppress fluctuating selection dynamics, depending on the type of interaction between the parasites. Enhanced fluctuating selection dynamics when coinfecting parasites increased fitness costs to the hosts were seen when the genetically specific parasite species was not harmful enough to induce fluctuating selection dynamics on its own. Considering the low to moderate fitness costs many parasite species induce to their hosts this type of interaction may have an important role in enhancing fluctuating selection dynamics in nature. When coinfections reduced parasite performance through resource competition, fluctuating selection dynamics became possible with very high levels of fitness costs to the host induced by the genetically specific parasite. However, for the majority of parasite species that induce low to moderate fitness costs to their hosts, resource competition in coinfections would reduce their potential to induce fluctuating selection dynamics. This was because, in our simulation, even higher fitness costs induced to the hosts by the genetically specific parasite than in the single host–single parasite interactions were needed. Therefore, the effect of competitive interactions on fluctuating selection dynamics may often be negative.

The exact mechanisms underlying the above effects of coinfections on fluctuating selection dynamics are not fully understood. In scenario one, the presence of the coinfecting parasite species and the increased fitness costs to the hosts in coinfections should lead to stronger control of the host population density by the parasites compared with the single host–single parasite interaction. Therefore, lower host densities that reduce the transmission potential of the genetically specific parasite can be expected. This would prevent a parasite that is not highly harmful to its hosts from spreading to a high and constant prevalence in the host population but instead inducing fluctuating selection dynamics (see the mechanism in Figure 3). Similarly, in scenario two, the presence of the coinfecting parasite that reduces the reproductive output of the genetically specific species would prevent it from driving host clones to extinction even if it was highly harmful to its hosts. However, the observed increase in the range of levels of fitness costs to the hosts induced by the genetically specific parasite leading to fluctuating selection dynamics would not be expected if only the mean changes in fitness costs to the hosts and parasite fecundity in coinfections contributed to epidemiological and coevolutionary dynamics. Thus, variation in both host and parasite performance when coinfections take place (i.e., some host individuals are infected with one and some with two parasite species) is likely to be important.

Because our results on the effects of coinfections on epidemiological and coevolutionary dynamics are likely to be explained by their impact on the ability of the genetically specific parasite to spread in the host population and to drive host clones to extinction also other ecological/environmental factors that modify host and parasite performance could have conceptually similar effects. For instance, resource availability and ambient temperature are well known to induce within‐population variation in host and parasite traits (see Brown et al., 2000; Guinnee & Moore, 2004; Krist et al., 2004; Paull & Johnson, 2011; Seppälä et al., 2008). To our knowledge, however, the potential effects of such factors on fluctuating selection dynamics have not been examined. Thus, our model gives the first insights on how ecological/environmental factors may affect fluctuating selection dynamics when host and parasite performance in the two coevolving populations is affected by external factors. The effects of other factors than coinfections should, however, be modeled separately. This is because changes in factors such as resource availability and ambient temperature are likely to follow different spatial and temporal dynamics than the epidemiology of coinfecting parasites that we modeled in this study.

Although the role of ecological/environmental factors on fluctuating selection dynamics in host–parasite interactions has not been examined before this study, earlier theoretical work has considered the combined effects of epidemiological and coevolutionary dynamics in host and parasite populations (e.g., Gokhale et al., 2013; MacPherson & Otto, 2018). Those studies suggest epidemiological dynamics to suppress coevolutionary cycles. However, our study, as well as the model by Lively (2010b) that was used as a basis for our simulation, both demonstrate fluctuating selection dynamics although they allow epidemiological dynamics. Additionally, most theoretical studies on host–parasite coevolution, including ours, assume host and parasite populations to be completely mixed and thus the encounters between the interacting partners to be random. Certain host and parasite types may, however, be clustered spatially, which could modify infection dynamics. For example, the aggregation of hosts into family groups can suppress coevolutionary fluctuations (Greenspoon & Mideo, 2017). Thus, also other deviations from random encounters such as vertical transmission could be important. Therefore, we argue that future studies should not only examine the possible effects of new factors on host–parasite coevolution but also their combined effects. We find it especially relevant to investigate how factors that can enhance fluctuating selection dynamics (e.g., certain coinfections) interact with factors that are known to suppress it (e.g., epidemiology, parasite transmission among relatives).

## CONFLICT OF INTERESTS

We declare we have no competing interests.

## AUTHOR CONTRIBUTION

**Otto Seppälä:** Conceptualization (lead); Formal analysis (lead); Funding acquisition (lead); Methodology (equal); Visualization (lead); Writing‐original draft (lead). **Curtis M. Lively:** Formal analysis (supporting); Methodology (supporting); Writing‐review & editing (equal). **Jukka Jokela:** Formal analysis (supporting); Methodology (supporting); Writing‐review & editing (equal).

## Data Availability

No data were used.
